# Clinical impact of cardiovascular magnetic resonance in evaluation for possible arrhythmogenic right ventricular dysplasia/cardiomyopathy

**DOI:** 10.1186/1532-429X-17-S1-Q53

**Published:** 2015-02-03

**Authors:** Andrew D Choi, Sujata M Shanbhag, Peter Kellman, Christine Mancini, Marcus Y Chen, Andrew E Arai, Patricia W Bandettini

**Affiliations:** 1 Advanced Cardiovascular Imaging Laboratory, National Heart, Lung and Blood Institute, National Institutes of Health, Bethesda, MD, USA; 2 Division of Cardiology, Medstar Washington Hospital Center, Washington, DC, USA

## Background

This study examined the impact of CMR on clinical management in patients with undergoing evaluation for arrhythmogenic right ventricular dysplasia/cardiomyopathy(ARVD/C).

## Methods

Patients referred for assessment of ARVD/C were evaluated. Using 2010 ARVD/C Task Force criteria, clinical history, family history, ECG and other test results were evaluated with and without CMR findings to determine definite, borderline or possible ARVD/C. CMR included assessment of right ventricular(RV) size, function, and regional wall motion(RWM). For alternative diagnoses, tissue characterization and late gadolinium enhancement were routinely performed. Qp:Qs was performed when intracardiac shunt was suspected by the supervising physician.

## Results

311 consecutive patients (mean age 45±14 years, 53% male) were included. Prior to CMR, patients were classified as definite (n=1), borderline (n=1) or possible ARVD (n=18, Table). After CMR, 6(2%) were diagnosed with definite ARVD/C and underwent defibrillator implantation, 5(2%) were classified as borderline ARVD/C, and 9 (3%) remained possible ARVD/C. 51(16%) had alternative diagnoses (Figure/Table), resulting in a management change: 6(1.9%) patients had intracardiac shunt, 36(11.5%) had another cardiomyopathy or RV overload state, and 9(2.8%) had other diagnoses. 76(24%) had RV enlargement alone with normal RV function and absent RWM by CMR while 164(53%) without other major criteria had normal RV function, size, and RWM.

**Figure 1:**
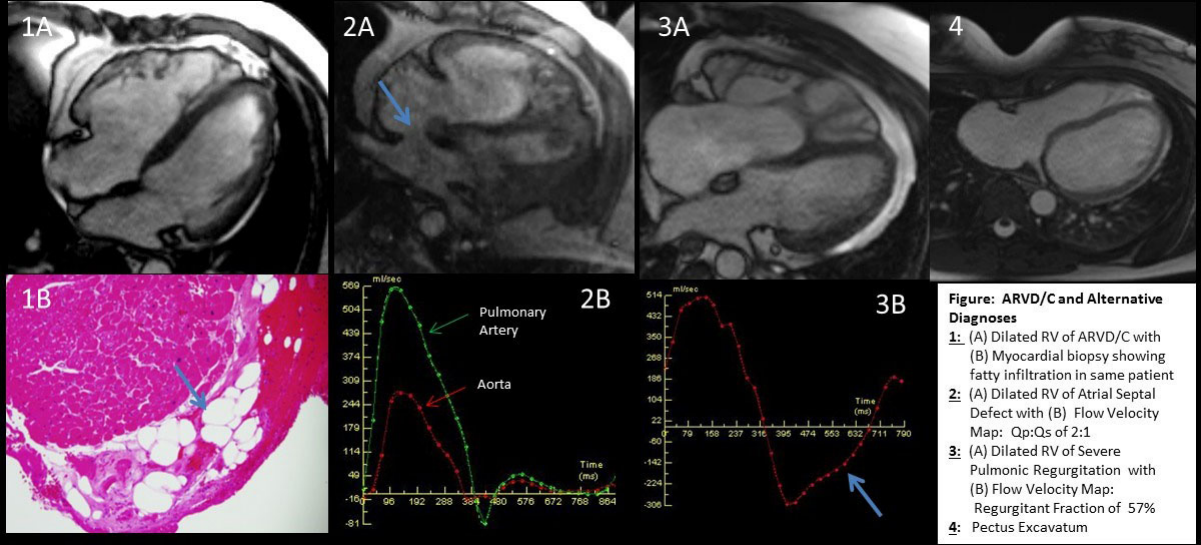


**Table 1:** Clinical Impact of CMR on Diagnosis of ARVD/C vs. Alternative Diagnoses

n=311	2010 Guidelines without CMR Findings, n (%)	2010 Guidelines with CMR Findings, n (%)
Definite Criteria for ARVD/C	1 (0.3)	6 (2)

Borderline Criteria for ARVD/C	1 (0.3)	5 (2)

Possible Criteria for ARVD/C	18 (5.8)	9 (3)

Patients with 1 or no Minor Criteria, not meeting 2010 Guidelines Definition of \"Definite\", \"Borderline\" or \"Possible\"	291 (93.6)	51 (16) Alternate Diagnosis* 76 (24) RV Enlargement Alone** 164 (53) Normal RV***

* Alternative Diagnosis Resulting in Change in Management included 6 (1.9%) Intracardiac shunts, other Cardiomyopathy or RV Overload State 36 (11.5%), or Other Diagnosis 9 (2.8%)

**RV Enlargement Alone with normal RV Function and Regional Wall Motion

*** Normal RV Function, Size and Regional Wall Motion by CMR

## Conclusions

CMR impacted clinical management by contributing to the diagnosis of definite or borderline ARVD/C in 4% of patients and by excluding the presence of significant RV dysfunction, enlargement, and RWM in over half of patients. CMR identified important alternative diagnoses in 16%.

## Funding

No conflicts of interest to disclose.

